# Parallel import mechanisms ensure the robust nuclear localization of actin in *Drosophila*

**DOI:** 10.3389/fmolb.2022.963635

**Published:** 2022-08-19

**Authors:** Péter Borkúti, Ildikó Kristó, Anikó Szabó, Csaba Bajusz, Zoltán Kovács, Zsuzsánna Réthi-Nagy, Zoltán Lipinszki, Tamás Lukácsovich, Sven Bogdan, Péter Vilmos

**Affiliations:** ^1^ Eötvös Loránd Research Network (ELKH), Biological Research Centre, Szeged, Hungary; ^2^ Doctoral School of Multidisciplinary Medical Science, University of Szeged, Szeged, Hungary; ^3^ Biological Research Centre, Institute of Biochemistry, MTA SZBK Lendület Laboratory of Cell Cycle Regulation, Eötvös Loránd Research Network (ELKH), Szeged, Hungary; ^4^ Brain Research Institute, University of Zürich, Zürich, Switzerland; ^5^ Department of Molecular Cell Physiology, Institute of Physiology and Pathophysiology, Philipps-University, Marburg, Germany

**Keywords:** actin, nucleus, nuclear transport, importin, *Drosophila*

## Abstract

Actin, as an ancient and fundamental protein, participates in various cytoplasmic as well as nuclear functions in eukaryotic cells. Based on its manifold tasks in the nucleus, it is a reasonable assumption that the nuclear presence of actin is essential for the cell, and consequently, its nuclear localization is ensured by a robust system. However, today only a single nuclear import and a single nuclear export pathway is known which maintain the dynamic balance between cytoplasmic and nuclear actin pools. In our work, we tested the robustness of the nuclear import of actin, and investigated whether the perturbations of nuclear localization affect the viability of the whole organism. For this aim, we generated a genetic system in *Drosophila,* in which we rescued the lethal phenotype of the null mutation of the *Actin5C* gene with transgenes that express different derivatives of actin, including a Nuclear Export Signal (NES)-tagged isoform which ensures forced nuclear export of the protein. We also disrupted the SUMOylation site of actin, suggested earlier to be responsible for nuclear retention, and eliminated the activity of the single nuclear import factor dedicated to actin. We found that, individually, none of the above mentioned manipulations led to a notable reduction in nuclear actin levels and thus, fully rescued lethality. However, the NES tagging of actin, together with the knock out of its importin, significantly reduced the amount of nuclear actin and induced lethality, confirming that the presence of actin in the nucleus is essential, and thereby, over-secured. Supporting this, we identified novel nuclear importins specific to actin, which sheds light on the mechanism behind the robustness of nuclear localization of actin, and supports the idea of essentiality of its nuclear functions.

## Introduction

Since its discovery, eukaryotic actin had been considered to perform its manifold functions exclusively in the cytoplasm, but recent recognition of the existence of prokaryotic actin-like proteins and nuclear actin in eukaryotes have opened new perspectives on the field. It is now clear, that in the nucleus of eukaryotic cells functionally active monomers, filament formation and movement of chromatin represent an ancient actin world (Moore and Vartiainen 2017; Plessner and Grosse, 2019). In addition, nuclear microfilament-associated motor proteins as well as branching and severing factors, all of which are eukaryotic developments of the microfilament system, are also present in the cell nucleus (Bajusz et al., 2018). In light of these findings it is not surprising, that nuclear actin has been found to participate in all fundamental nuclear functions, such as transcription, RNA processing, nuclear export, chromatin remodeling, replication, and DNA damage repair (Kristó et al., 2016; Misu et al., 2017; Kelpsch and Tootle 2018; Percipalle and Vartiainen 2019; Ulferts et al., 2021).

The discovery that eukaryotic actin is not an exclusively cytoplasmic protein and that it plays diverse roles in the nucleus, argues for the existence of tightly regulated, active nuclear transport which can control protein levels in the two cell compartments, thereby maintaining a dynamic balance between cytoplasmic and nuclear actin pools. Actin contains two putative nuclear export sequences (NES) (Wada et al., 1998) that can be recognized by the export factor CRM1/exportin1. One of the models of actin export suggests that sumoylation on K284 blocks access to the NES-1 sequence resulting in sumoylated actin being retained in the nucleus (Collier et al., 2000; Hofmann et al., 2009; Alonso et al., 2015). The evolutionarily highly conserved sumoylation site of actin (MKCD) corresponds to a canonical sumoylation consensus sequence (Ψ-K-x-D/E), in which the lysine residue is the site for SUMO protein attachment. The second model states that the RanGTP-bound export factor, exportin-6 removes actin from the nucleus with the help of the actin-binding protein, profilin (Stüven et al., 2003). Although, in contrast to multiple NES motifs, actin lacks any canonical nuclear localization (NLS) sequence, it has been shown that the import factor, importin-9, is responsible for the nuclear import of actin monomers in complex with the NLS-bearing, actin-binding cofactor, cofilin (Nishida et al., 1987; Dopie et al., 2012). These findings together highlight the complexity of nuclear transport of actin, and suggest that its nucleo-cytoplasmic shuttling is influenced by many factors and mechanisms, such as the activity of importins and exportins, as well as the amounts and availability of monomeric actin, profilin or cofilin (Ulferts et al., 2021).

Based on these, it is feasible to conclude that the nuclear presence of actin is essential for the cell. However, this is only an assumption, since no effort has yet been made to directly investigate the importance of its nuclear localization or to provide evidence for the robustness of its nuclear localization mechanisms. To explore the biological significance of nuclear actin, the activity of the protein should be inhibited specifically in the nucleus. However, actin undertakes indispensable tasks in the cytoplasm and, as ongoing research indicates, it interacts with the same binding partners in the nucleus and in the cytoplasm (Kristó et al., 2016; Percipalle and Vartiainen 2019). Therefore, the faithful separation of the nuclear activity from the cytoplasmic functions does not seem possible at the moment.

In this work we aimed to investigate the biological significance of nuclear actin through testing the robustness of its nuclear localization. To this aim, we used *Drosophila* as a genetic model system which enabled us to investigate this question at the level of the whole organism.

## Materials and methods

### Fly stocks and husbandry

All *Drosophila* stocks were maintained and crosses were carried out on standard cornmeal, yeast, sucrose *Drosophila* medium at 25°C unless otherwise noted. Stocks number 3605 (*w* ^1118^), 279 (*w* ^1118^; MKRS, P{*hs*-FLP}86E/TM6B, *Tb* ^1^), 31450 (*w* ^1118^; Dp(1;3)DC146, PBac{DC146}VK00033), and 25709 (*y* ^1^, *v* ^1^, P{*nos*-phiC31\int.NLS}X; P{CaryP}attP40) were obtained from Bloomington *Drosophila* Stock Center (BDSC). Stocks number 123887 (*w* ^1118^, P{RS3}*Act5C* ^CB−6034–3^), 126186 (*w* ^1118^, P{RS5}*Act5C* ^5−SZ−3675^) and 205564 (*y* ^1^, *w* ^67c23^; P{*w* ^+mC^ = GSV6}GS13460/TM3 *Sb* ^1^, *Ser* ^1^) were obtained from Kyoto Stock Center. Stocks *w*; SM6b/*Sp*; Df/TM3 P(Δ2-3) and *w* ^1118^; CyO, Δ2-3/*Sp*; Df(3L)BSC386/TM3 *Sb* ^1^, *Ser* ^1^ were kindly provided by Rita Sinka (Univ. of Szeged). Injection of *Drosophila* embryos from stock No. 25709 were carried out by the BRC *Drosophila* Injection Facility (BRC, Szeged).

To analyze embryonic and larval lethal phenotypes of *Act5C* null mutant stocks, flies were first fed yeast-paste in egg-laying cages for 1 day. To synchronize egg laying and thereby the age of the progeny, flies were transferred to fresh black agar medium every hour. After the third transfer, 200 individual eggs were aligned by hand in groups of 100 on a fresh black agar plate. The following day the number of hatched and unhatched eggs were counted to quantify embryonic lethality. To monitor larval development and lethality, the same number of eggs were collected with the same method, and their development was monitored for 72 h. The GFP signal was detected and imaging was performed with a Leica MZ FLIII fluorescence stereo microscope.

### Generation of null mutations

To create the *Act5C* null mutation, the stocks, crossing schemes and instructions described by the DrosDel project (Ryder et al., 2004; Ryder et al., 2007) were applied. Stock number 279 was used as an FLP source which expresses flippase through a heat shock-inducible promoter. RS element activation was induced by incubation at 37°C for 1 h. Animals of the two, activated RS stocks were crossed and a second heat shock incubation was performed on them. The female offsprings with red/white variegating eyes were crossed individually with FM7c,*w* ^a^ balancer chromosome-bearing male flies to establish stable *Act5C* null mutant stocks.

In order to delete the *Ipo9* gene*,* P-element carrying stock No. 205564 was crossed with flies carrying transposase source on their second chromosome balancer. The mutagenesis was performed using the standard *Drosophila* P-element-mediated mutagenesis method (Hummel and Klämbt 2008; Ou 2013).

For the molecular characterization of mutant lines, genomic DNA was prepared according to the following protocol: 30 anesthetized flies were collected in a sterile Eppendorf tube. Animals were ground with 400 µL Buffer A (100 mM Tris-Cl (pH 7.5), 100 mM EDTA, 100 mM NaCl, 0.5% SDS), then incubated for 30 min at 65°C. Incubation was followed by the addition of 800 µL Buffer B [200 ml potassium acetate (5 M), 500 ml lithium chloride (6 M)], brief vortexing and another incubation for 1 h at room temperature (RT). Next, the samples were centrifuged for 15 min at ×18,000 *g* and 4°C. 1 ml of supernatant was transferred into a new Eppendorf tube and 600 µL isopropanol was added. Samples were centrifuged at RT for 15 min at ×18,000 *g*. Pellets were washed with 70% ethanol, air-dried and resuspended in 100 μL TE + 0.5 µL RNase (Thermo Fisher Scientific, EN0531).

Mutant lines were validated by PCR amplification of the genes. Primers Act5CFup2 (5′-CCA​GTT​GCG​GAG​GAA​ATT​CTC), Act5CRev2 (5′-ATG​ATG​CGA​TTA​AAG​TGC​CGT), and Pry4 (5′- CAA​TCA​TAT​CGC​TGT​CTC​ACT​CA) were used to amplify the mutant *Act5C* gene. The primers RanBP9_Fw1 (5′-TTG​TAC​TGA​GCA​GGC​TTA​ACA) and RanBP9_Rev2 (5′-GGT​TTG​CAT​TCT​AAA​AGC​CTC​G) were used to determine the break points of the deletion in *Ipo9*. PCRs were performed according to the standard protocol for DreamTaq polymerase (Thermo Fisher Scientific, EP0701). Sequencing of the PCR products (and all sequencings) was performed with the primers used in the PCR reactions, by Eurofins Genomics TubeSeq Service.

### RNA isolation, cDNA synthesis and PCR reaction

For the further validation of the null alleles, total RNA was isolated from L1-stage (*Act5C*) or L3-stage (*Ipo9* and wild type) larvae. Thirty animals were collected per genotype in 1X PBS. Samples were homogenized in 250 µL TRIzol (Thermo Fisher Scientific, 15596026), followed by the addition of 70 µL nuclease-free water. Samples were mixed by inverting the Eppendorf tubes several times, and incubate them on RT for 5 min. Next, 70 µL chloroform was added to each sample, followed by vigorous vortexing. After incubation for 5 min at RT, samples were centrifuged at 4°C, for 10 min at ×18,000 *g* (Eppendorf 5430 R). The aqueous phase, containing the total RNA, was transferred to a new Eppendorf tube and equivalent amount of isopropanol was added. After 10 min incubation at RT, samples were centrifuged at 4°C, for 10 min at ×18,000 *g*. The supernatant was discarded and the RNA pellet was washed with 500 µL of 75% ethanol. Samples were centrifuged at 4°C, for 5 min at ×6,800 *g*, and the pellets were air-dried. Dry pellets were dissolved in 25 µL of nuclease-free water.

To eliminate DNA contamination, the total RNA samples were treated with DNase I (Thermo Fisher Scientific, EN0521) according to the manufacturer’s instructions. cDNAs were prepared using random hexamers with the RevertAid First Strand cDNA Synthesis Kit (Thermo Fisher Scientific, K1622) according to the manufacturer’s instructions. Reverse transcribed cDNAs were used as template in the PCR reactions. The *Act5C* transcript was amplified using Act5C_UTRF2 (5′- CAG​TCA​TTC​CTT​TCA​AAC​CG) and ExonR1 (5′-AAC​CAG​AGC​AGC​AAC​TTC) primers. To test for *Ipo9* mRNA production, primers Exon1F (5′-TAA​GCA​AGC​CAT​CAT​CGA​G) and Exon2R (5′- GGT​TCT​CCA​CGT​AAC​GAG) were applied. The PCR reaction was performed with Q5 High-Fidelity DNA polymerase (New England Biolabs, M0491S). The size of amplicons was determined with GeneRuler 100 bp DNA Ladder (SM0241).

### Creating Act5C expressing transgenic lines

For the generation of the transgenes expressing modified Act5C proteins under the regulation of the endogenous promoter, the 7.2 Kbp genomic region around *Act5C* was PCR amplified with the 5′for(Gateway) forward (5′-AGG​ATC​CGG​GGA​CAA​GTT​TGT​ACA​AAA​AAG​CAG​GCT​ACG​ATC​GAG​CAA​CGA​ACT​TGA​G and 3′rev(Gateway) reverse (5′-GGG​GAC​CAC​TTT​GTA​CAA​GAA​AGC​TGG​GTC​GGG​GAC​CACT​TT​GTA​CAA​GAA​AGC​TGG​GTT​AAA​CCG​CTC​AAG​GTGCT​A​CG) primers using Q5 High-Fidelity DNA polymerase (New England Biolabs, M0491S). The sequences encoding the NES (5′-AAT​GAA​TTA​GCC​TTG​AAA​TTA​GCA​GGT​CTT​GAT​AT​CAAC​AAG​ACA) and V5 (5′-ATG​AAT​GGT​AAG​CCT​ATC​CCT​AAC​CCT​CTC​CTC​GGT​CTC​GAT​TCT​ACG) epitope tags were cloned after the START codon of *Act5C* using overlapping PCR reactions and the following primers: 5′rev (5′-tac​gcg​tca​tcG​TTT​AAA​CAC​TTG​CGG​TGC​ACA​ATG​GAG​G), 3′for (5′-tac​gcg​tAG​GAT​CGC​TTG​TCT​GGG​CAA​G), Actin-seq1 (5′-GTA​CTT​TGG​TAG​ACC​AGC​GCA​G), Actin-seq2 (5′-TTT​GAC​CGA​CTA​CCT​GAT​GAA​G), Bam-Mlu-Not-for (5′-GAT​CCC​ATG​ACG​CGT​CAT​GGC), Bam-Mlu-Not-rev (5′-GGC​CGC​CAT​GAC​GCG​TCA​TGG), Stop-for (5′-Cgcggccgca), Stop-rev (5′-CGC​GTG​CGG​CCG​CG), NESfor (5′-CAA​TGA​ATT​AGC​CTT​GAA​ATT​AGC​AGG​TCT​TGA​TAT​CAA​CAA​GAC​ATA​A), NESrev (5′-CGC​GTT​ATG​TCT​TGT​TGA​TAT​CAA​GAC​CTG​CTA​ATT​TCA​AGG​CTAA​TT​CAT​TG), V5for (5′-cAA​TGG​TAA​GCC​TAT​CCC​TAA​CCC​TCT​CCT​CGG​TCT​CGA​TTC​TAC​GTA​A), and V5rev (5′-CGC​GTT​ACG​TAG​AAT​CGA​GAC​CGA​GGA​GAG​GGTT​AG​GGA​TAG​GCT​TAC​CAT​TG).

The *Act5C* genomic regions expressing modified Act5C proteins were inserted into pBluescript II SK vector from which they were subcloned with the BamHI + XhoI enzymes into the pAttB *Drosophila* transformation vector (*Drosophila* Genomics Resource Center #1420). The FLAG encoding dsDNA oligo (FLAGfor 5′-CGC​GCG​ACT​ACA​AGG​ACG​ACG​ATG​ACA​AGG​GAA and FLAGrev 5′-CGC​GTT​CCC​TTG​TCA​TCG​TCG​TCC​TTG​TAG​TCG) was inserted downstream from the NES and V5 tags with the help of a unique MluI restriction site. The constructs were sequence verified and injected into *Drosophila* embryos carrying an attP integration site on their second chromosome at cytological location 25C6 (BDSC stock #25709).

### Rescue experiments

All rescue experiments were performed on normal *Drosophila* food media at 25°C. Crossing schemes were planned and carried out by standard *Drosophila* mating methods. Virgin females were always collected from null mutant stocks, while males were collected from the transgenic “rescue stocks”. Male offsprings of the different genotypes were categorized by phenotypic markers.

## Molecular cloning and DNA constructs

### Cloning of Act5C-K285R expressing constructs

To acquire the *Act5C* gene at a manageable size for mutagenic PCR, a region of the previously amplified *Act5C* genomic fragment (see: *Creating Act5C expressing transgenic lines*) was excised from the Actin5C-pAttB construct with FastDigest HindIII restriction enzyme (Thermo Fisher Scientific, FD0504). The product with the right fragment size was gel-extracted with NucleoSpin Gel and PCR Clean-up Kit (Macherey-Nagel, 740609). The clean fragment was subcloned into pBSK + vector using HindIII and T4 DNA ligase (New England BioLabs, M0202) enzymes. The nucleotide substitution at K285 of *Act5C* was carried out on this construct with QuickChange II Site-Directed Mutagenesis Kit (Agilent, 200523) using Act5C_K285R_Fw (5′-CCT​ACA​ACT​CCA​TCA​TGA​GGT​GTG​ATG​TGG​ATA​TCC​G), and Act5C_K285R_Rev (5′-CGG​ATA​TCC​ACA​TCA​CAC​CTC​ATG​ATG​GAG​TTG​TAG​G) (mutated bases are underlined) primers designed with the QuickChange Primer Design online tool of Agilent (Novoradovsky et al., 2005). Isolation of the plasmid (and all plasmid isolation) was performed with High-Speed Plasmid Mini Kit (Geneaid, PD3000). After sequencing, the right construct with the desired point mutation and the Act5C-pAttB construct were digested with the HindIII enzyme. The fragments of the correct size were extracted from agarose gel, and the Act5C-K285R-pAttB construct was created by ligation with T4 DNA ligase (New England BioLabs, M0202). The right clone was validated by sequencing. The Act5C-K285R-pAttB construct was injected by the BRC *Drosophila* Injection Facility (BRC, Szeged) into embryos carrying an AttP integration site on their second chromosome at cytological location 25C6 (BDSC stock #25709). Three transgenic fly stocks were generated via standard *Drosophila* mating methods.

For the experiments in cultured *Drosophila* cells, the mutagenesis reaction was performed on the Act5C-pAGW construct (Kristó et al., 2017) with the aforementioned mutagen primers and was validated by sequencing.

### Gateway cloning of importin CDS

The cDNAs of importin candidates obtained from the *Drosophila* Genomics Resource Center (DGRC) Gold Collection were PCR amplified with Q5 High-Fidelity DNA polymerase (New England Biolabs, M0491S) using the following gateway cloning primers (underscore marks the gene specific region):

Arts_GW_Fw2 5′-GGG​GAC​AAG​TTT​GTA​CAA​AAA​AGC​AGG​CTT​CAC​CAT​GGA​GGC​AGC​TAT​TCT​GGA, Arts_GW_Rev2 5′-GGG​GAC​CAC​TTT​GTA​CAA​GAA​AGC​TGG​GTC​GCT​CAG​GGC​TTG​CAC​ATA​ATT​GA, Moleskin 3_GW_Fw2 5′-GGG​GAC​AAG​TTT​GTA​CAA​AAA​AGC​AGG​CTT​CAC​CAT​GGA​GG​C​ACA​AAA​ACT​CAC, Moleskin_GW_Rev2 5′-GGG​GAC​CAC​TTT​GTA​CAA​GAA​AGC​TGG​GTC​GCT​GGA​GCC​GAA​CTT​AA​A​CGA​CG, TRN_GW_Fw2 5′-GGG​GAC​AAG​TTT​GTA​CAA​AAA​AGC​AGG​CTT​CAC​CAT​GCA​GCG​AAA​CAT​GAT​GAT, TRN_GW_Rev2 5′-GGG​GAC​CAC​TTT​GTA​CAA​GAA​AGC​TGG​GTC​GCT​GTG​CGT​GAA​CTG​ACG​CAG​GG.

Entry clones were generated by recombining the PCR products with Gateway BP Clonase II Enzyme mix (Thermo Fisher Scientific, 11789020) into the pDONOR221 plasmid. After sequencing the clones, the DNA regions encoding the importins were subcloned with Gateway LR Clonase II Enzyme mix (Thermo Fisher Scientific, 11791020) into split YFP tagging vectors (Gohl et al., 2010). Actin was labelled at the amino-terminus with the N-terminal fragment of YFP (nYFP), while bait proteins were tagged both at the N- and C-terminal ends with the C-terminal fragment of YFP (cYFP).

### Cloning of Act5C for *in vitro* pull-down experiments

To generate GST-tagged actin, the CDS of Act5C was cloned into pGEX-6P-1 vector (GE Healthcare) in frame with the N-terminal GST-tag. The CDS was PCR amplified with the Act5C_GST_Fw1 5′-CCC​GGG​TCG​ACT​CGA​CAT​GTG​TGA​CGA​AGA​AGT​TGC, and Act5C_GST_Rev1 5′-GAT​GCG​GCC​GCT​CGA​TTA​GAA​GCA​CTT​GCG​GTG​CA primers using Q5 High-Fidelity DNA polymerase (New England Biolabs, M0491S). The resulting Act5C-pGEX-6P-1 construct was validated with DNA sequencing.

### Cell culturing

The S2R + *Drosophila* cell line was maintained at 25°C in Schneider’s *Drosophila* medium (Lonza) complemented with 10% Fetal Bovine Serum (Whittaker) and 1% antibiotics (Penicillin-Streptomycin, Gibco). The Effectene Transfection Reagent Kit (Qiagen, 301425) was used to transfect cells according to the manufacturer’s instructions. For the live imaging of cells, 8 × 10^6^ cells/35 mm glass bottom Petri dishes (Cell E&G, GBD00001-200) were used.

For the BiFC importin screen, split YFP-tagged prey and bait expressing constructs were co-transfected with pMT-Gal4 vector (DGRC #1042) which expresses the Gal4 protein under the control of an inducible metallothionein promoter. Two days after transfection, CuSO_4_ was added to the cells in 1 mM final concentration to induce protein expression. After 2 hours, the CuSO_4_ containing medium was replaced with 3 ml of fresh medium and the interaction was visualized with a Zeiss LSM800 confocal microscope (×20 and ×63 OIL objective lenses).

### Immunohistochemistry

All steps of the immunostaining experiments were carried out at RT unless otherwise noted. For larval salivary gland dissections, egg laying was synchronized as described above. The eggs were allowed to develop for 3 days, then L3 stage larvae were collected and dissected. For the immunostaining of fly ovaries, females were kept on yeast paste together with males for 3 days prior to dissection. Cultured *Drosophila* S2R + cells were transfected as described above. Dissected salivary glands and ovaries, and cultured cells were fixed in 4% paraformaldehyde-PBS followed by a washing step with PBS. Fixed tissues and cells were permeabilized with PBT (PBS + 0.1% Triton X-100) for 20 min. Non-specific reactions were blocked with PBT-N solution (PBT, 1% BSA, 5% FCS) for 1 h. Next, samples were incubated overnight (O/N) with anti-FLAG primary antibody (M2, 1:1000, Sigma-Aldrich, F1804) at 4°C. Next, samples were incubated overnight (O/N) with the primary antibody at 4°C. Anti-FLAG antibody (M2, 1:1000, Sigma-Aldrich, F1804) was used to stain salivary glands, and the anti-HA primary antibody (1:800, Merck, H3663) was used for cultured cells. After washing three times with PBT for 10 min, samples were incubated in PBS containing Alexa488 coupled anti-mouse secondary antibody (1:600, Biotium, 20010-1), and DAPI (0.2 μg/ml, Sigma-Aldrich, D9564) for 1 h in the dark. After a final wash with PBS, stained glands were transferred to microscope slides, mounted in 20 µL Fluoromount-G medium (Thermo Fisher Scientific, 00-4958-02), and imaged with a confocal microscope (Olympus Fluoview FV1000, 60x OIL objective lens (salivary glands), and Zeiss LSM800 ×63 OIL objective (ovaries and cultured cells).

### Database search, quantification of pixel intensities and statistical analysis of data

The nuclear-cytoplasmic fluorescence intensity ratio was measured with Image J software (Schneider et al., 2012). ROIs in the nucleus and the cytoplasm were drawn by hand. In every experiment 3-6 larval salivary glands, with a minimum of 5 cells/gland, were measured. Mean value and standard deviation (SD) were calculated with Microsoft Excel, while unpaired t-tests for statistical analysis and graph creation were performed with GraphPad Prism 9.

## *In vitro* pull down experiments

### Recombinant protein purification

For protein purification, the Actin5C^R63D^-pET16b construct was transformed into *E. coli* SixPack (Lipinszki et al., 2018) competent cells. A single transformed colony was grown in 50 ml of standard liquid growth medium until the culture’s density reached an OD600 of 0.6–0.8. Protein expression was induced O/N with the addition of 0.5 mM IPTG. Induced bacteria were washed and lysed by sonication in 10 ml of Lysis Buffer [100 mM NaH_2_PO_4_xH_2_O pH 8.0, 150 mM NaCl, 5 mM imidazole, 0.5% N-Lauroylsarcosine sodium salt (Sigma-Aldrich, L5125)] supplemented with 1.5 ml EDTA free 7X PIC (Merck, 11873580001). The lysate was centrifuged for 15 min with ×10,000 *g* (Eppendorf 5430 R) at 4°C. HisPur Cobalt Resin beads (Thermo Fisher Scientific, 89964) were washed with 2 ml of Lysis Buffer supplemented with 0.5 mg/ml BSA, for 5 min, at 4°C, two times. Next, beads were centrifuged at 500 x *g* for 5 min, at 4°C. Following the centrifugation step, 10 ml of lysate supplemented with 50 µL BSA (100 mg/ml) was added to the beads, and the mixture was incubated for 2 h at 4°C on a rotating platform. After incubation, the beads were washed 3 times, for 20 min with 5 ml of Wash Buffer (100 mM NaH_2_PO_4_xH_2_O pH 8.0, 300 mM NaCl, 20 mM Imidazol, 0.2% N-Lauroylsarcosine sodium salt) and centrifuged at ×500 *g* for 5 min at 4°C and. Finally, His-Act5C^R63D^ protein (bait) immobilized to beads was stored in 250 µL storage buffer (5 ml ×10 PBS, 28.7 ml 87% glycerol, 16.3 ml UP water for 50 ml storage buffer solution) at -20°C.

### *In vitro* transcription and translation of prey proteins

Coupled *in vitro* transcription and translation of ^35^S-labelled importin candidates was carried out using the TNT Quick Transcription/Translation System (Promega, L1170) as described earlier (Karman et al., 2020). Purified His-Act5C^R63D^ was used as bait while His-GFP-FLAG served as negative control. Briefly, for each IVTT reaction, 90 ng of T7-promoter-regulated cDNA template (Tnpo-pOT2, Tnpo-SR-pOT2 RanBP9-pOT2, RanBP11-pOT2, Cadmus-pOT2, Ketel-pOT2, Msk-pHY22, Artemis-pHY22) was used in 15 µL reaction volume containing 11 µL TNT master mix (Promega), 0.33 µL PCR enhancer (Promega), 0.54 µL MBq ^35^S-methionine (Perkin Elmer), 0.33 µL U RNAsin (Promega) and 0.33 µL 50x EDTA-free protease inhibitor cocktail (Roche). The reaction was carried out at 30°C for 1 h, followed by centrifugation at 4°C, for 5 min at ×17,000 g. From the supernatant 0.25 µL was used as input, while 7.3 µL was used for each *in vitro* pull down assay.

### *In vitro* his-pull down assay and autoradiography

The *in vitro* pull-down assay was based on the method described earlier (Karman et al., 2020). His-GFP-FLAG (negative control) or His-Act5C^R63D^ (bait) immobilized onto Cobalt beads were washed with Pull-down Wash Buffer 1 (PDWB1) (50 mM HEPES pH 7.5, 150 mM NaCl, 2 mM MgCl_2_, 1 mM EGTA, 1 mM DTT, 0.1% Triton X-100, 5% glycerol, 20 mM imidazole) and mixed with the IVTT-produced ^35^S-labeled prey proteins in Binding Buffer (PDWB1 supplemented with 1x EDTA-free PIC (Roche) and 0.5 mg/ml BSA) for 1.5 h at 4°C. Beads were washed three times with PDWB1, followed by 3 washes with Pull-Down Wash Buffer 2 (PDWB1 supplemented with 50 mM NaCl and 0.1% Triton X-100). Finally, beads were collected in 1x SDS Sample Buffer and boiled for 5 min. Proteins were separated by 10% SDS-PAGE and blotted onto PVDF membrane (Merck-Millipore, IPVH00010). The membrane was stained with Ponceau S (Sigma) to visualize the amount of bait proteins, then scanned, dried and directly used for autoradiography. Exposure to autoradiography film (Kodak, Biomax MS) was carried out at -80°C using Low energy transcreen (Kodak Biomax LE).

## Results

### A modular, two-component genetic system enables the suppression of nuclear actin activity in the fruit fly

To analyze the robustness of the nuclear localization of actin, and thereby examine the biological relevance of this localization, we decided to generate a mutant *Drosophila* line with decreased nuclear actin. To achieve our goal, we aimed to tag the protein with an additional, CRM1-recognised NES sequence of the cAMP-dependent protein kinase inhibitor alpha (PKIA) (Güttler et al., 2010). We chose this NES, because it has been shown to be effective in the fly (Kazgan et al., 2010), and therefore ensures constant removal of actin from the nuclei while actin’s cytoplasmic functions remain intact (Bajusz et al., 2018). There are six actin coding genes in *Drosophila*, whose protein coding regions are highly similar thus, the *in situ* tagging of one of the genes with the CRISPR/Cas9 method was not an option. Therefore, we constructed a modular genetic system in which one actin gene is deleted and different transgenes ensure the expression of various forms of the corresponding actin protein.

Out of the six actin isoforms in *Drosophila,* only two, Act5C and Act42A, collectively called cytoplasmic actins, are expressed in all cells at all times during development (Gelbart and Emmert 2013). The other four actin proteins are active mostly in muscle sarcomeres of certain organs at specific developmental stages. Earlier reports showed that of the two cytoplasmic actins, only *Act5C* is critical for the survival of the organism (Wagner et al., 2002). In addition, regulation of the *Act5C* gene has been thoroughly described (Vigoreaux and Tobin 1987; Bond-Matthews and Davidson 1988; Chung and Keller 1990), and its 7.2 Kbp size makes it an ideal subject for transgenic manipulations. Taking these into account, we chose *Act5C* for the experiments.

**FIGURE 1:**
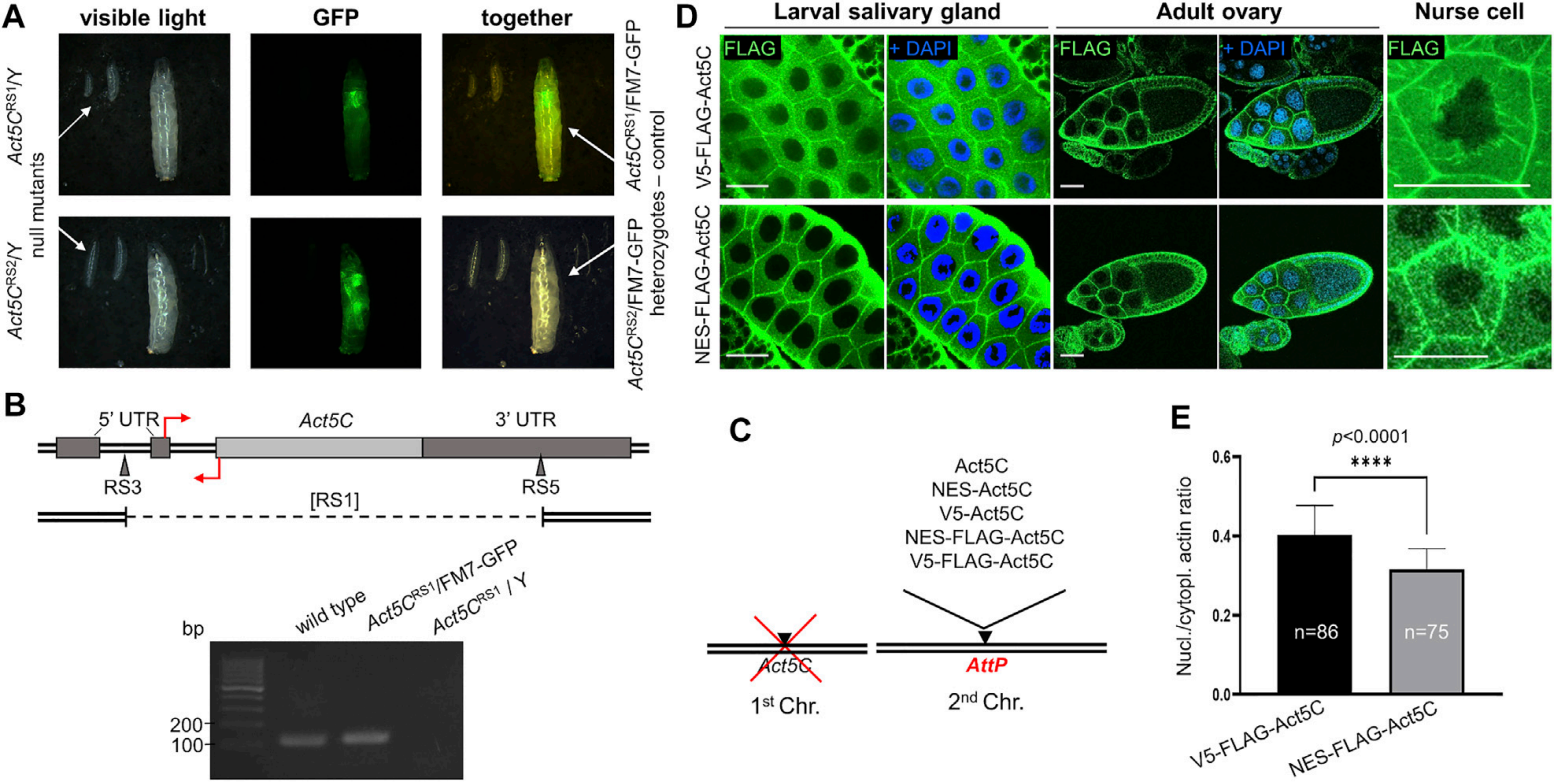
Rationale of the experimental system used to investigate nuclear actin. **(A)** Both *Act5C* null alleles (RS1 and RS2) cause 100% developmental arrest in the first larval stage in homo- and hemizygous form as compared to their heterozygote, third-instar female siblings of the same age marked with a GFP expressing balancer chromosome (FM7-GFP). **(B)** Genomic organization of *Drosophila Act5C* gene. Protein coding (light gray rectangle) and noncoding (dark grey rectangles) exons are shown. The insertion sites for the RS3 and RS5 transposons used to generate a deletion are marked with triangles. Deleted segment in the [RS1] allele is represented by dashed line under the map. Primers for RT-PCR are indicated by red arrows. Forward primer binding in the 3′ UTR is located above; reverse primer binding behind the translation START site is located below the DNA. Gel (below) shows RT-PCR products of 136 bp using the primers shown above. **(C)** The modular genetic system used in this study consists of the null mutation of *Act5C* on the first chromosome, and various transgenes expressing different forms of actin under the regulation of *Act5C* inserted into the same genomic position on an autosome. **(D)** Immunostaining of larval salivary gland cells and adult ovaries for actin (V5-FLAG-Act5C) and NES-tagged actin (NES-FLAG-Act5C) proteins (green) expressed from transgenes. Nuclei are visualized by DAPI staining (blue). Scale bar, 50 μm. **(E)** Quantification of the immunostaining of salivary glands in **(D)**. Unpaired *t-*test was used for statistical analysis, ****p < 0.0001. Error bars represent standard deviation. “*n”* is the number of cells examined.

In order to set up a genetic system to reduce the amount of nuclear actin in the fly, first we generated a null mutant allele for *Act5C* by inducing site directed recombination between “Rearrangement Screen” type P-elements (RS3 and RS5 elements) (Golic and Golic 1996; Beumer et al., 1998) residing in the *Act5C* locus. Two independent candidate null mutants carrying the same deletions, were identified by their restored red eye-color in the screen. The flies were used to establish two stocks that both showed only slight embryonic lethality (28% compared to 11% normal embryonic lethality in the wild type control), but exhibited slow and disoriented movement, 100% developmental arrest (Figure 1A) and subsequent 100% lethality at the first larval stage in homo- and hemizygous forms. The lethality was fully rescued with a duplication containing the *Act5C* region (Dp(1;3)DC146) confirming that the observed phenotypic changes are due to the deletion of *Act5C* (not shown). We named these mutant lines *Act5C* ^RS1^ and *Act5C* ^RS2^. The RS1 line was used in all following experiments and will be referred to as *Act5C* null throughout the text. To unambiguously verify that the deletion is specific to *Act5C*, we sequenced the corresponding genomic region in *Act5C* ^RS1^, and found that the majority of the coding region, including the entire single protein coding exon, is missing in the mutant (Figure 1B). To further validate the RS1 allele, RT-PCR was performed on total RNA of L1-stage larvae. The amplified DNA fragment of the expected size (136 bp) was present in the wild type (*w* ^1118^) and *Act5C* heterozygous null mutant control animals, but no Act5C transcript was detected in *Act5C* ^RS1^ homozygous null larvae (Figure 1B).

For the generation of transgenic stocks expressing modified actin proteins, the *Act5C* gene was PCR amplified, *in vitro* modified and inserted into the genome. To ensure the native regulation and identical expression of all transgenes, the genomic section covering the region between the upstream and downstream neighboring genes of *Act5C* was amplified, and site directed integration using the same genomic AttP landing platform site (Venken et al., 2006; Bischof et al., 2007) was applied. The protein coding sequence in the transgene was modified by inserting the following epitope tags after the START codon: NES (to ensure continuous export of actin from the nucleus), V5 (to control the NES tag because they are of the same size (16 amino acids), and FLAG (for the detection of the protein) (Figure 1C). To check whether protein expression from the transgenes was normal, larval and adult tissues expressing FLAG epitope tagged Act5C isoforms were immunostained and the protein expression was documented by confocal microscopy. We observed strong expression of Act5C isoforms in all transgenic lines, suggesting that protein expression is normal in these animals (not shown). We also compared the subcellular distribution of V5-FLAG-Act5C and NES-FLAG-Act5C in larval salivary glands and adult ovaries, and found that they are the same and correspond to the already known, wild type localization (Figure 1D). In the ovary, both actin forms accumulate at the cortex of all types of cells, and in the nurse cells of the 10th developmental stage egg chamber, they form the cytoplasmic filopodia-like cables tethering the nuclei into the center of the cell (Figure 1D).

To analyze the effect of the NES tag on nuclear actin level, we measured the fluorescence intensity in the nucleus and the cytoplasm of larval salivary gland cells with Image J software, and calculated the ratio between them. Approximately 25% decrease in signal intensity was observed in NES-FLAG-Act5C expressing animals compared to V5-FLAG-Act5C expressing larvae (Figure 1E). These results led to the conclusions that transgenes ensure wild type expression of Act5C in the transgenic stocks, the tagging of Act5C does not interfere with its localization, and that the NES tag fused to Act5C reduces but does not fully eliminates the protein from the nucleus.

### Increased nuclear export has no effect on viability

**FIGURE 2:**
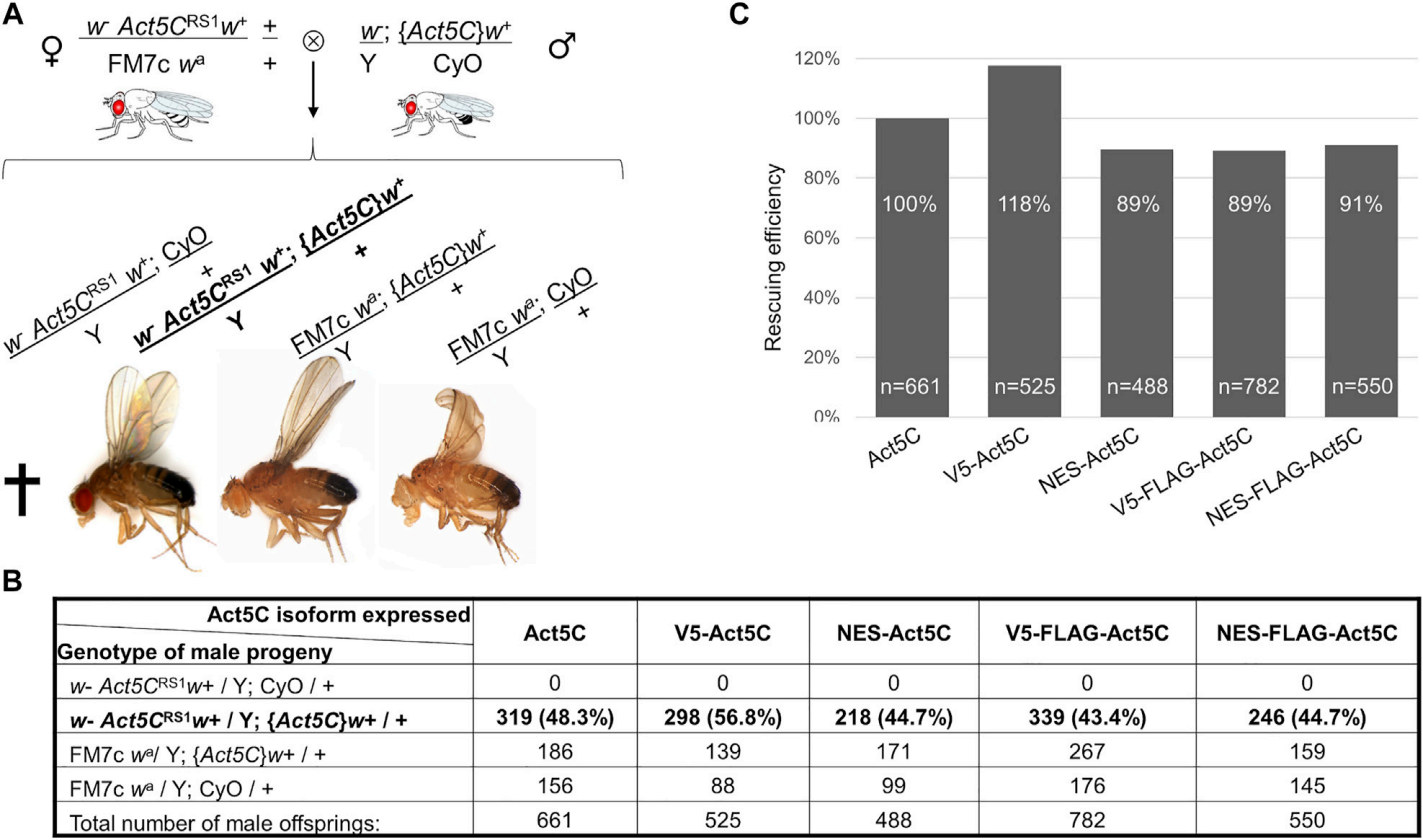
NES tagging of actin has no effect on viability. **(A)** General scheme of the rescue experiments, and the genotype categories and phenotypes of male F1 progeny. *Act5C* ^RS1^ symbolizes the null allele of the *Actin5C* gene, {*Act5C*} symbolizes Actin5C expressing transgene, cross indicates lethality. The genotype of rescued males is in bold. **(B)** The number of male F1 progeny of the different genotypes in five rescue experiments. The genotype of rescued males is in bold. **(C)** Bar chart presentation of the data in **(B)**. The rescue effect (percentage ratio of the rescued category) of wild type actin (Act5C) was considered as 100%, and the rescuing efficiency (percentage ratio value of the rescued category) of other actin forms was compared to it. “*n”* is the total number of male offspring in the given rescue cross.

We next tested if reduced amount of nuclear actin due to the NES sequence had any impact on the viability of the flies. Remarkably, the lethal phenotype of the *Act5C* null allele was completely rescued by the transgenes expressing differently tagged Act5C isoforms. Because *Act5C* is located on the first chromosome, the number of male progeny that carry the *Act5C* null allele on their single X chromosome was counted, and the ratio between the different genotype categories (Figure 2A) was calculated. To distinguish male progeny that inherited their Y chromosome from their mother bearing an additional Y chromosome (XXY) and their X chromosome from their father (XY), the first chromosome in all of the *Act5C* transgenic lines was marked with a recessive mutation of the *yellow* (*y* ^*-*^) gene causing apparent yellow body color in males (Figure 2A). The expression of wild type actin resulted in very effective rescue which we considered 100% and used as a reference in the subsequent experiments. Surprisingly, there was no significant difference between the percentages of control males and their male siblings rescued by the *NES-Act5C* transgene (Figure 2B) which indicates normal rescuing efficiency (Figure 2C). This suggests that a ∼25% decrease in nuclear actin level does not affect its essential biological functions, and has no effect on viability.

### Sumoylation at K285 is not necessary for nuclear functions

**FIGURE 3:**
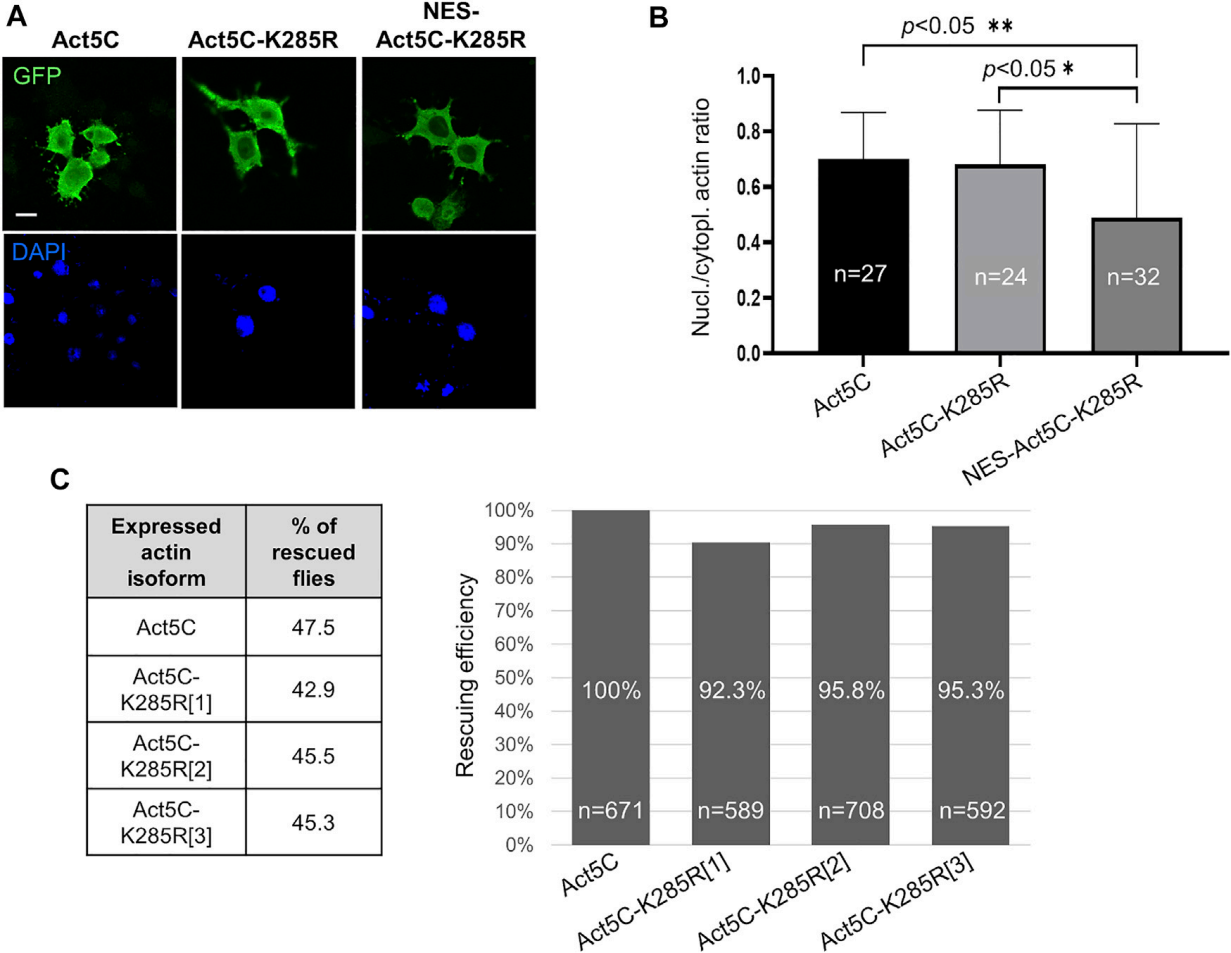
Sumoylation at K285 does not contribute significantly to nuclear retention of actin in *Drosophila*. **(A)** Immunostaining with anti-GFP antibody of *Drosophila* S2R + cells expressing GFP-tagged wild type (Act5C), non-sumoylatable (Act5c-K285R) and NES-tagged + non-sumoylatable (NES-Act5C-K285R) actin proteins (green). Nuclei are visualized by DAPI staining (blue). Scale bar, 50 μm. **(B)** Quantification of immunostaining in **(A)**. “*n”* is the number of cells examined. Unpaired *t-*test was used for statistical analysis. *p*-values: ** = 0.0048 and * = 0.0155. Error bars represent standard deviation. **(C)** Bar chart presentation of the data of the rescue experiments with the non-sumoylatable form of actin. Three independent transgenic *Drosophila* lines which express Act5C-K285R under the endogenous *Act5C* promoter (Act5C-K285R[1-3]) have been used in three parallel experiments. The percentage ratio of the flies rescued with wild type actin (Act5C) was considered as 100% rescue. The percentage of rescuing efficiency was calculated by comparing the percentage ratio of the rescued category obtained with the Act5C-K285R forms to the 100% rescue. “*n”* is the total number of male offspring in the given rescue cross.

According to the literature, sumoylation of actin acts as a nuclear retention signal due to the masking of the NES motif by the conjugated SUMO protein (Hofmann et al., 2009). The lysine residue in the evolutionarily conserved sumoylation motif of actin can be found in the amino acid position 285 in *Drosophila*. We aimed to investigate whether the lack of sumoylation at K285 reduces nuclear actin level, and thereby the viability of the flies. To this end, we replaced the lysine with another positively charged residue, arginine (K285R), which results in a non-sumoylatable form of actin (Hofmann et al., 2009). GFP-labelled Act5C-K285R mutant protein was expressed first in cultured *Drosophila* S2R + cells (Figure 3A) and the ratio between nuclear and cytoplasmic fluorescence intensities was calculated. No significant difference was observed between the ratios of nuclear and cytoplasmic fluorescence intensities of GFP-Act5C and GFP-Act5C-K285R however, GFP-NES-Act5C-K285R showed a 27% reduction of this value (Figure 3B). The lower fluorescence ratio in the case of GFP-NES-Act5C-K285R is in agreement with the previous result obtained with NES-FLAG-Act5C (Figure 1C), and is obviously due to the effect of the NES sequence attached to actin and not to the mutation of the sumoylation site.

In parallel to the experiments with cultured cells, we generated three independent transgenic *Drosophila* lines that express Act5C-K285R under the regulation of the endogenous *Act5C* promoter (*Act5C* ^K285R^[1-3]). The ratios between the different genotype categories of surviving male progeny were about the same when the *Act5C* ^RS1^ null mutant allele was complemented with wild type Act5C (47.5%) or Act5C-K285R (43%, 45.5%, and 45.3% with Act5C^K285R^[1], Act5C^K285R^[2], and Act5C^K285R^[3], respectively) (Figure 3C) therefore, the rescuing efficiency of the Act5C-K285R protein is similar to wild type actin (Figure 3C). This result supports the observation obtained with transfected S2R + cells, and argues against the idea that sumoylation of actin at K285 serves as a robust nuclear retention signal in *Drosophila*.

### Depletion of Ipo9 leads to reduced nuclear actin levels, and decreases viability when actin is NES-tagged

**FIGURE 4:**
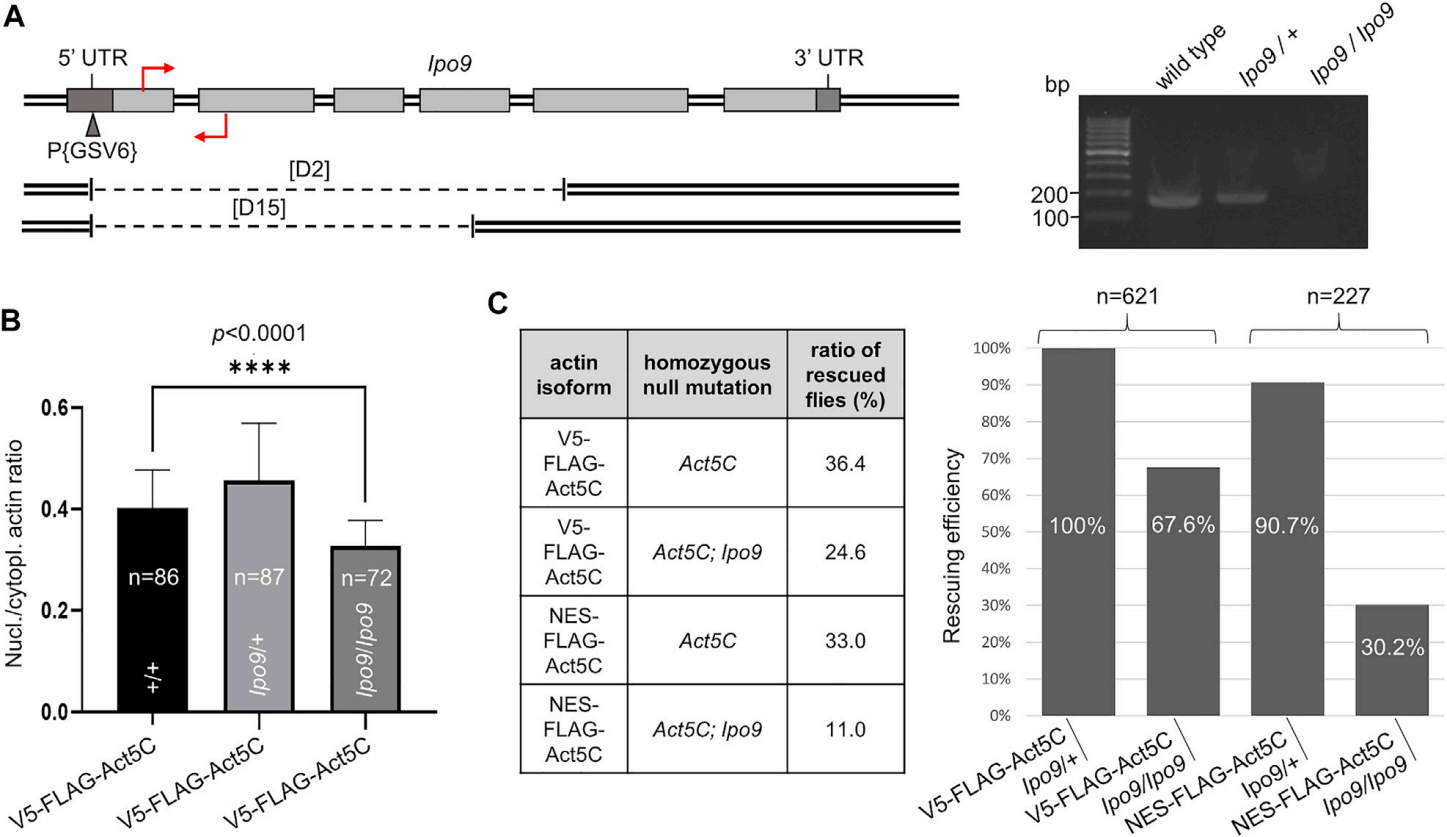
Lack of Ipo9 reduces nuclear actin level but has negative effect on viability only in combination with the NES tag. **(A)** The exon-intron structure of the *Drosophila Ipo9* gene. Protein coding (light grey rectangles) and noncoding (dark grey rectangles) exons are shown. The insertion site for the P{GSV6} element in the 5′UTR is marked with a triangle. Deleted segments in *Ipo9* ^D2^ and *Ipo9* ^D15^ are shown under the map by dashed lines. Primers for RT-PCR are indicated by red arrows. Forward primer binding in the first exon is located above; reverse primer binding in the second exon is located below the DNA. Gel shows RT-PCR products of 200 bp using the primers shown. **(B)** Bar chart presentation of the quantification of nuclear actin (V5-FLAG-Act5C, green) expressed from a transgene in wild type animals, and in heterozygote (*Ipo9/+)* and homozygote (*Ipo9/Ipo9) Ipo9* null mutant background. “*n”* is the number of cells examined. Unpaired *t-*test was used for statistical analysis, *p*-value: * < 0 0001. Error bars represent standard deviation. **(C)** Summary of the rescue experiments with actin (V5-FLAG-Act5C) and NES-actin (NES-FLAG-Act5C) in *Ipo9* null background. The percentage of rescuing efficiency was calculated as follows. The percentage ratio of the flies rescued with V5-FLAG-Act5C in heterozygous *Ipo9* background (*Ipo9*/+) was considered as 100% rescue, and the percentage ratio of the rescued category obtained in the other crosses was compared to it. “*n”* is the total number of male offspring hatched in the given rescue cross.

Ipo9 (also known as RanBP9), the *Drosophila* orthologue of the vertebrate importin of actin, is encoded by the *Ipo9* gene located on the third chromosome of the fruit fly. It was previously shown that the deletion of *Ipo9* decreases nuclear actin levels (Sokolova et al., 2018; Palacios et al., 2021). Therefore, we tested whether the combination of an *Ipo9* deficient background with NES-tagged actin expression can significantly reduce nuclear actin level and, as a consequence, viability. We generated a null allele for *Ipo9* by inducing imprecise excision of a P-element residing in the 5′UTR of the gene. Twenty-five candidate mutant lines were recovered based on the loss of their red eye color. Two of them carried deletions of 2140 and 1825 bp that extended from the P-element insertion site to the interior of the fifth or fourth coding exon, respectively (Figure 4A). The lines were named *Ipo9* ^D2^ and *Ipo9* ^D15^. Since the deletions eliminated the putative ATG start codon and the majority of the protein coding region, they can be considered as physically verified nulls. To unambiguously confirm that D2 is a bona fide null allele, RT-PCR was performed on total RNA of L3-stage larvae. The amplified DNA fragment of the appropriate size (200 bp) was detected in the wild type (*w* ^1118^) and *Ipo9/+* heterozygous null mutant control animals, but no *Ipo9* transcript was amplified from *Ipo9/Ipo9* homozygous null larvae (Figure 4A).

Both mutant lines showed 100% sterility in males and females, but no visible phenotypic changes could be observed. This phenotype was in good agreement with recently published results about *Ipo9* null (Palacios et al., 2021). *Ipo9* ^D2^ flies were used in all subsequent experiments, and the allele is referred to as *Ipo9* null.

FLAG-tagged actin protein was expressed from the rescue transgenes in wild type animals and in flies, which were null mutants for *Ipo9* in either heterozygous or homozygous form. Immunostaining of larval salivary gland cells with a FLAG antibody, and calculation of the ratio between nuclear and cytoplasmic fluorescence intensities (Figure 4B) revealed no significant difference between the wild-type and *Ipo9* null heterozygous cells. However, fluorescence intensity ratios showed an 18% reduction (0.4 vs. 0.33 nuclear/cytoplasmic actin ratio) when the *Ipo9* null allele was in homozygous form (Figure 4B). This suggests that complete lack of the Ipo9 importin decreases nuclear actin levels but does not fully eliminate actin from the nucleus.

In order to use the *Ipo9* null mutation in homozygous form in the rescue experiments, we established an *Act5C* ^RS1^; *Ipo9* ^D2^ double mutant stock and also introduced the *Ipo9* null allele into the rescue lines expressing V5-FLAG-Act5C or NES-FLAG-Act5C. In these rescue experiments, males carrying the *Ipo9* null allele in homo- or heterozygous form were also present among the progeny of a single cross, enabling accurate comparison of the viability of the two genetic conditions. Rescue of the *Act5C* null mutation showed that similarly to previous results, the NES tag alone has no significant effect on viability, the ratio of rescued flies was 33% (vs. 36.4% in the control) when the *Ipo9* null allele was present in heterozygous form, which means 90.7% rescuing efficiency for NES-FLAG-Act5C (Figure 4C). The loss of both copies of the *Ipo9* gene reduced the viability by 33% (24.6 vs. 36.4%) when rescuing with wild type, V5-tagged actin, confirming that the complete loss of Ipo9 causes only partial lethality. In contrast to this, when the rescue was performed with the NES-tagged actin protein and on homozygous *Ipo9* null mutant background, they together decreased viability by 67% (11.0 vs. 33.0%) and reduced the rescuing efficiency by 70% (30.2%) (Figure 4C). This suggests that the combined effect of the NES tag and the *Ipo9* null mutation on actin’s nuclear localization strongly impairs viability. However, these effects together are still not sufficient to fully exclude actin from the nucleus and cause 100% lethality.

### Multiple importins are responsible for the robust nuclear localization of actin

**FIGURE 5:**
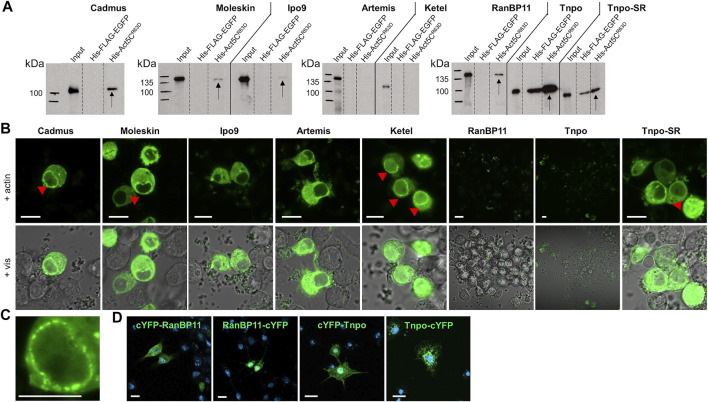
Multiple importins contribute to the robust nuclear localization of actin. **(A)** Pull-down experiment with importins and actin. Arrows mark the importins pulled down by actin. **(B)** Split-YFP screening for importins interacting (green) with actin in transfected live S2R + cells. Red arrowheads mark the punctate pattern of the fluorescent signal at the nuclear envelope. Pictures show the results obtained with C-terminally tagged importins. Vis—visible light. Scale bar, 50 μm. **(C)** Magnification showing the punctate pattern at the nuclear membrane. Scale bar, 50 μm. **(D)** Visualization of protein expression. Transfected S2R + cells were immunostained with anti-HA antibody (green) for N-terminally (cYFP-RanBP11 and cYFP-Tnpo) and C-terminally (RanBP11-cYFP and Tnpo-cYFP) tagged RanB11 and Tnpo proteins. DAPI staining, blue. Scale bar, 100 μm.

The observation that in the absence of Ipo9 importin actin is still able to enter the nucleus, suggests that parallel mechanisms secure the nuclear localization of actin. This implies that mechanisms or factors other than Ipo9 should be involved in the nuclear import of actin. Therefore, we sought to identify additional importins that are responsible for the nuclear translocation of actin in *Ipo9* null mutant animals. First, we searched the fly genome for putative importin genes and identified 4 alpha and 16 beta importins (Table 1). We selected the nine importins of the beta importin family, including the already known actin importin, Ipo9, for which the full-length cDNA was available, and used them to perform *in vitro* pull-down experiments. The results showed that, in addition to the already known interaction with Ipo9, Cadmus, Moleskin, RanBP11, Tnpo, and Tnpo-SR are able to bind the monomeric form of actin (Act5C^R63D^) *in vitro* (Figure 5A). This provides additional evidence that these nuclear import proteins can physically interact with actin, presumably in order to transport it into the nucleus. In the case of Tnpo and Tnpo-SR background binding to His-FLAG-EGFP was also observed, but the intensity of these unspecific reactions was weaker than their binding to actin (Figure 5A). Therefore, we consider the interaction of Tnpo and Tnpo-SR with actin to be specific. The finding, that Artemis, Ketel and Karyopherin β3 failed to pull down actin in our assay, might be due to the limitations of the *in vitro* method used, and it does not rule out their role as import factors of actin.

**TABLE 1:** Importins of *Drosophila melanogaster*. References: [1] Virágh et al., 2012; [2] Mason et al., 2009; [3] Mason et al., 2002; [4] Máthé et al., 2000; [5] Kimura and Imamoto, 2014; [6] Lippai et al., 2000; [7] Boeynaems et al., 2016; [8] Mosca and Schwarz, 2010; [9] VanKuren and Long, 2018; [10] Sokolova et al., 2018; [11] Palacios et al., 2021; [12] Bhattacharya and Steward 2002; [13] Betrán and Long 2003.

Gene symbol	Cg number	Gene name	Also known as	Closest human homolog	References
ALPHA importins					
Kap-α1	CG8548	Karyopherin α1	importin α1, imp α1, Dα1, impα1	KPNA6	(Virágh et al., 2012; Mason et al., 2009; Mason et al., 2002; Máthé et al., 2000)
Pen	CG4799	Pendulin	importin α2, imp-α2, mushroom body miniature B, mbmB, oho31	KPNA2	(Virágh et al., 2012; Mason et al., 2009; Mason et al., 2002; Máthé et al., 2000)
Kap-α3	CG9423	Karyopherin α3	imp-α3, importin α3, Importin-α3, impα3, imp α3	KPNA4	(Virágh et al., 2012; Mason et al., 2009; Mason et al., 2002; Máthé et al., 2000)
αKap4	CG10478	α Karyopherin-4	divergent fourth α-importin-like gene	-	(Mason et al., 2009; Mason et al., 2002)
BETA importins					
Cse1	CG13281	Chromosome segregation 1	Cas, Dcas, l(2)k03902, CAS/CSE1 segregation protein	CSE1L	(Kimura and Imamoto, 2014; Lippai et al., 2000)
Fs(2)Ket	CG2637	Female sterile (2) Ketel	Ketel, MRE26, importin β, importin-β, imp-β	KPNB1	(Kimura and Imamoto, 2014; Lippai et al., 2000; Boeynaems et al., 2016; Mosca and Schwarz, 2010)
Apl	CG32165	Apollo	RanBP4	IPO4	(Boeynaems et al., 2016)
Arts	CG32164	Artemis	RanBP5	IPO5	(VanKuren and Long, 2018)
Karyβ3	CG1059	Karyopherin β3	l(3)j3A4, l(3)82CDd, l(3)j7E8, RanBP6	IPO5	(Lippai et al., 2000)
msk	CG7935	moleskin	Dim-7, CIP-61, Imp7, Corkscrew Interacting Protein-61, Dim7, RanBP7	IPO7	(Kimura and Imamoto, 2014; Lippai et al., 2000)
Ipo9	CG5252	Importin 9	RanBP9	IPO9	(Sokolova et al., 2018; Palacios et al., 2021)
Impβ11	CG33139	Importin beta11	RanBP11, importin-β11, Imp11, CG8212	IPO11	(Kimura and Imamoto, 2014; Lippai et al., 2000; Boeynaems et al., 2016; Mosca and Schwarz, 2010)
cdm	CG7212	cadmus	importin 13, sd-5, Imp13, cdm	IPO13	(Kimura and Imamoto, 2014)
emb	CG13387	embargoed	Crm1, dCRM1, Exportin, XPO1, l(2)k16715	XPO1	(Kimura and Imamoto, 2014; Lippai et al., 2000)
Ranbp21	CG12234	RanBP21	Exp5, Exportin-5, Exp-5, Exportin 5	XPO5	(Lippai et al., 2000)
CG8219	CG8219			TNPO1	(Lippai et al., 2000)
Tnpo	CG7398	Transportin	Trn, dTRN, Imp beta2	TNPO1, TNPO2	(Lippai et al., 2000; Boeynaems et al., 2016)
Tnpo-SR	CG2848	Transportin-Serine/Arginine rich	Trn-SR, TNPO3	TNPO3	(Lippai et al., 2000)
Ntf-2	CG1740	Nuclear transport factor-2	DNTF-2, Ntf2, l(1)G0428, l(1)G0086, l(1)G0337, NTF2R	NUTF2	(Bhattacharya and Steward 2002; Betrán and Long 2003)
CG10950	CG10950		Imp beta-like	TNPO3	

To verify the newly identified interactions between actin and its putative importins, we performed a Bimolecular Fluorescence Complementation (BiFC) screen in cultured *Drosophila* S2R + cells. The BiFC technology is based on the tagging of two interacting proteins with non-fluorescent fragments of a fluorescent protein (e.g. YFP). The close proximity of the two proteins allows the reconstitution of the functional fluorescent protein, which enables the direct visualization of the protein-protein interaction *in vivo* (Cabantous et al., 2005). We labelled the importins with a YFP fragment at the amino terminus and the carboxyl terminus as well, and tested both protein variants in the case of all importins. None of the tagged forms of Tnpo and RanBP11 gave a positive signal in the assay, and we failed to test Karyopherin β3 due to cloning difficulties. But beside Ipo9, Ketel, Cadmus, Moleskin, Artemis, and Tnpo-SR transportins exhibited clear interaction with actin (Figure 5B). Both N- and C-terminally tagged forms of Moleskin and Artemis showed positive reaction with actin, while in the case of the other importins (Ipo9, Ketel, Cadmus, Tnpo-SR) only C-terminal tagging resulted in a fluorescent signal. Interestingly, the nuclear fluorescent signal intensities of the different importins were not the same, which might reflect different binding stabilities. Two different cellular patterns of the fluorescent signal were observed in the experiment. Ipo9 and Artemis bound to actin showed homogenous distribution in the cytoplasm and the nucleus (except for the nucleolus) without any specific pattern. In contrast, in the case of Ketel, Cadmus, Moleskin, and Tnpo-SR not only a diffuse cytoplasmic and nuclear fluorescent signal was present, but also a punctuated accumulation at the nuclear envelope could be observed (red arrowheads in Figures 5B,C). Finally, we performed antibody staining to verify that the negative BiFC result obtained with RanBP11 and Tnpo are not due to the lack of protein expression. The experiment revealed that both the N-terminally and C-terminally tagged forms of both proteins are produced (Figure 5D). In the case of Ipo9, Cadmus, Moleskin and Tnpo-SR the interaction with actin could be confirmed by both *in vitro* and *in vivo* methods, therefore these importins most likely contribute to the dynamic and robust nuclear localization of actin. Based on these, we conclude that multiple nuclear importins can transport actin into the nucleus in *Drosophila*.

## Discussion

Nuclear localization and functions of actin are the subject of extensive research nowadays. However, its diverse and essential cytoplasmic tasks, the absence of a canonical NLS motif in the protein sequence, and the coincidence of its interaction partners and molecular mechanisms in the nucleus and the cytoplasm, hamper the separation of nuclear functions from cytoplasmic activity, and thereby the direct testing of the *in vivo* relevance of nuclear localization seems unachievable. To overcome this problem and gain deeper insight into the nuclear functions of actin, we created a *Drosophila* line in which a NES tag on the actin protein warrants forced nuclear export. Contrary to our expectations, the NES motif only partially decreased nuclear actin level and did not affect viability. This finding reveals that actin’s import system can effectively counterbalance an increase in export, and demonstrates the robustness of nuclear actin import. In addition, the mutation of the K285 amino acid implicated as a key for nuclear retention in mammalian cells, had no further impact on the nuclear localization and activity of NES-actin in *Drosophila*.

The size of the actin protein (42 kDa) is around the size threshold of 40 kDa for passive diffusion through the nuclear pore complex (Christie et al., 2016; Musser and Grünwald 2016), and dynamic nuclear translocation has been observed for actin (Dopie et al., 2012), which together suggest that actin is passively travelling into the nucleus. However, the concentration of actin in the cytoplasm is much higher than in the nucleus, and considering the abundance of the protein in the cytoplasm, fast passive diffusion could be compensated only by a constant and extremely active nuclear export, which is, obviously, an unlikely scenario. Fast passive diffusion into the nucleus is also hindered by the abundant amount of G-actin sequestering proteins in the cytoplasm, which is essential to prevent spontaneous actin polymerization (Pollard et al., 2000). In support of this, it was demonstrated that increasing the size of actin with GFP (27 kDa) or even double-GFP (52 kDa) does not affect the fast import rate of actin or its uneven distribution between the nucleus and the cytosol (Dopie et al., 2012). These observations strongly suggest that tightly regulated, highly effective active import mechanism is the primary mean of actin’s nuclear entry.

Until recently, only a single actin importin, Ipo9, had been identified in mammalian cells (Dopie et al., 2012), which had also been confirmed in *Drosophila* (Sokolova et al., 2018; Palacios et al., 2021). Interestingly, despite the fact that actin is not the only cargo of Ipo9—in mice it transports ribosomal proteins, histone H2B and heat shock protein hsp27 (Jäkel et al., 2002)—homozygous null mutant flies are viable with no phenotypic change. Although, there is no measurable lethality of the *Ipo9* null mutation in *Drosophila* (own observation, not shown), the complete loss of the importin should have some effect on viability. This idea was supported by the fact that both the NES tag and the lack of Ipo9 reduced nuclear actin levels to similar degrees, but viability was decreased only in the case of the *Ipo9* null mutation. Despite semi-lethality, nuclear actin levels are still high in the complete absence of Ipo9 function, which proves that actin utilizes additional mechanism(s) to enter the nucleus. In addition, even though the lethality effect of the *Ipo9* knock out and the extra NES tag showed a strong synergy, every fourth fly still survived, providing additional evidence that Ipo9 is not the exclusive importin of actin, and alternative mechanisms are also responsible for its nuclear localization.

**FIGURE 6:**
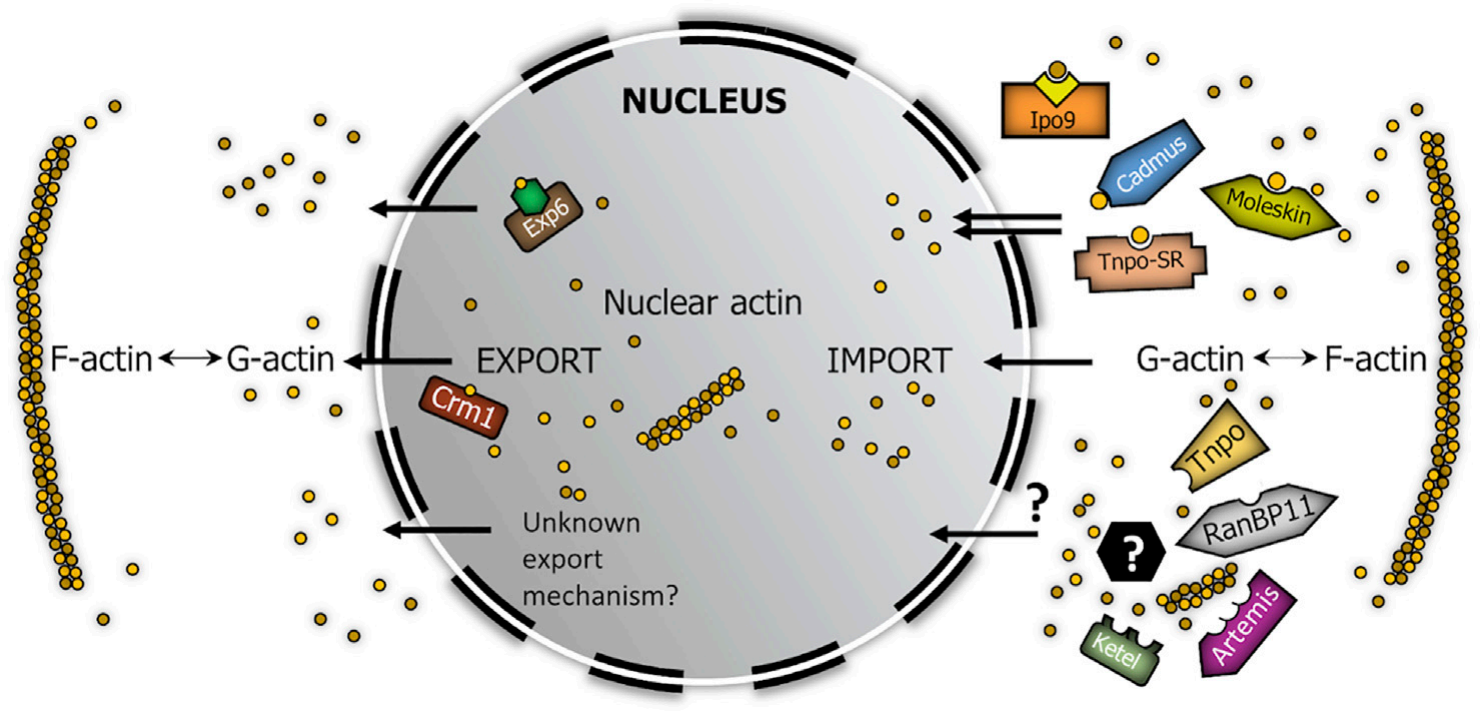
Model summarizing the robust nucleo-cytoplasmic transport of actin in *Drosophila*. Brown circles represent actin monomers. Yellow square in complex with Ipo9 represents cofilin, green hexagon in complex with Exp6 symbolizes profilin. Black hexagon with question mark symbolizes actin-binding import cofactor.

Based on these, we screened for new nuclear importins of actin with pull-down experiments and the BiFC technique. Cadmus, Moleskin, Tnpo-SR (IPO13, IPO7, and TNPO3 in human, respectively) interacted with actin both in *in vitro* and *in vivo* conditions therefore, we identified them as new nuclear import factors of actin in *Drosophila* (Figure 6). Four of the importins examined showed interaction only in the *in vitro* or in the *in vivo* test, which can be due also to differences between the two experimental systems. Ketel (KPNB1) and Artemis (IPO5) failed to pull down actin in our IVTT assay, but showed clear interaction with actin in the BiFC experiment. Moreover, they accumulated at the nuclear membrane in a punctate pattern (Figure 5B). It is likely that the lack of an *in vitro* interaction with actin in the case of these two importins is only due to the *in vitro* experimental system used (e.g., a co-factor is missing for binding), and these two importins actually participate in actin import. The non-polymerizable form of actin (Act5C^R63D^) was pulled-down by all of these importins in the co-immunoprecipitation experiments, which shows that actin is transported into the nucleus by all newly identified importins in monomeric form. The only exception from this could be Ketel and Artemis, which failed to pull down monomeric actin, but interacted with actin in living cells. Interestingly, RanBP11 and Tnpo pulled down actin *in vitro*, but they failed to interact with actin in the *in vivo* assay. However, the negative result in the BiFC experiment does not rule out the possibility that RanBP11 and Tnpo can still transport actin, it only means that none of the ends of these two proteins is proximal to the amino terminus of actin upon interaction.

Our aim was to demonstrate the existence of parallel import mechanisms behind the robust nuclear localization of actin. Because not all *Drosophila* beta importins have been tested in our screen, it is possible that there are even more import factors dedicated to actin than we have identified. Nonetheless, our work demonstrates the unambiguous existence of multiple actin importins which provides a good explanation for the high import rate of actin, which is similar to that of passive diffusion (Dopie et al., 2012). At the same time, our findings shed light on the mechanism through which the nuclear actin pool can so effectively overcome the impact of disturbing effects. On the whole, it is an obvious assumption that the evolution of parallel nuclear import mechanisms for actin suggests that actin’s nuclear localization is essential for the cell, which consequently strongly indicates essential nuclear functions for actin.

Although, the interaction assays we used do not reveal the direct or indirect nature of the interaction between actin and the given importin, but considering the lack of a canonical NLS motif in actin and the high number of monomeric actin binding factors (Sun et al., 1995; Pollard 2016), it is a feasible theory that the piggy-back mechanism described in the case of Ipo9-mediated nuclear transport of actin (Dopie et al., 2012) is a general phenomenon and is the key to the regulation of nuclear actin import.

The genetic system used in our work provides evidence for the indispensability of nuclear actin functions, and because NES-Actin expressing *Act5C*; *Ipo9* double null mutant flies show semi-lethality, it can also help the investigation of nuclear actin function at the organism level. The two different cellular patterns and the different nuclear intensities of the fluorescent YFP signal observed in the BiFC assay might reflect different binding stability and thus, a hierarchy between the importins, or they suggest different nuclear import mechanisms for actin. The hierarchy and interaction between the different actin import pathways, as well as the description of potential new export factors for actin are interesting issues to be resolved by future research.

## Data Availability

The original contributions presented in the study are included in the article/supplementary material, further inquiries can be directed to the corresponding author.
